# Testicular disorder of sexual development with cryptorchidism, penile hypoplasia and hypospadias in a giraffe (*Giraffa camelopardalis giraffa*)

**DOI:** 10.4102/jsava.v91i0.1971

**Published:** 2020-03-26

**Authors:** Janine Meuffels, Ilse Luther-Binoir, Willem Daffue, Francois Deacon, Emily P. Mitchell

**Affiliations:** 1 Department of Production Animal Sciences, Faculty of Veterinary Science, University of Pretoria, Onderstepoort, South Africa; 2 GEOsperm – Wildlife Reproduction and Biotechnology Services, Brits, South Africa; 3 Profetura – Alliance for Wildlife Conservation Breeding, Hamburg, Germany; 4 Kroonstad Animal Hospital, Kroonstad, South Africa; 5 Department of Animal, Wildlife and Grassland Sciences, University of the Free State, Bloemfontein, South Africa; 6 Department of Paraclinical Sciences, Faculty of Veterinary Science, University of Pretoria, Onderstepoort, South Africa

**Keywords:** cryptorchidism, disorder of sexual development, *Giraffa camelopardalis*, hypospadias, penile abnormality

## Abstract

Disorders of sexual development (DSD) in wild mammals are rarely described. A male South African giraffe (*Giraffa camelopardalis giraffa*) was identified with bilateral cryptorchidism. The testes were intra-abdominal, smaller and less ovoid than in normal male giraffes. The right testis was situated more cranially than the left and connected to a longer deferent duct with normal ampullae. One distended vesicular gland filled with mucoid material was identified. A short penis, situated in the perineal area, was directed caudally and presented hypospadias. Histologically, testicular hypoplasia was present; the epididymis tubules contained no spermatozoa and the deferent duct and vesicular gland were inflamed. The blood testosterone concentration was 16.27 nmol/L and oestrone sulphate concentration was 0.03 ng/mL. The aetiology of the abnormalities is unknown.

## Introduction

The reproductive system of the male giraffe is similar to that of other ruminants. The penis is situated in the ventral midline and opens cranially. It is fibro-elastic and has a sigmoid flexure and a rounded, laterally flattened head of the penis (glans penis) with an attached urethral process. The testes are ovoid (10 cm – 14 cm × 6 cm – 8 cm without the epididymis) and suspended into the scrotum with a vertical long axis. The accessory genital glands consist of prominent ampullae, paired non-lobulated vesicular glands (seminal vesicles), ovoid bulbo-urethral glands and the prostate, with a lobular disseminated part (pars disseminata), situated in the wall of the pelvic urethra (Hall-Martin, Skinner & Hopkins 1978).

Disorders of sexual development (DSD), formerly termed ‘intersex’, are congenital abnormalities in the development of chromosomal (abnormalities in XX or XY sex chromosome number or structure), gonadal (testicular, ovarian and ovotesticular DSD, previously termed true hermaphrodite, or dysgenesis) or phenotypic (male or female with abnormalities of tubular and external genitalia) sex. In gonadal sex abnormalities, the chromosomal and gonadal sex differ, although in phenotypic sex abnormalities, the chromosomal and gonadal sex are congruent, but the genitalia deviate (Hughes 2008; Lyle 2007).

In XY males, the activation of the sex-determining region Y chromosome gene (SRY) initiates testis development. Testis secretions promote the masculinisation of the internal and external male genitalia. Anti-Mullerian hormone (Mullerian inhibiting substance) causes regression of the paramesonephric duct (Mullerian duct, which forms the female internal genitalia in XX animals), although testosterone drives the development of the male tubular tract, accessory glands and external genitalia, and together with insulin-like factor 3 (INSL3) the descent of the testes. Possible XY, SRY+ testicular DSDs include hypospadias, cryptorchidism, testicular agenesis and hypoplasia, segmental aplasia of the epididymis, deferent ducts and penile hypoplasia (Lyle 2007).

Cryptorchidism is a condition whereby one or both testes and the associated structures are absent in the scrotum because of a failure of the testes to descend from the pre-natal abdominal position. The descent of testis includes three phases: abdominal testis translocation, transinguinal testis migration and inguinoscrotal testis migration. Abdominal testis translocation is dependent on INSL3 and only partially on testosterone, although the inguinoscrotal migration is androgen reliant (Amann & Veeramachaneni 2007). This type of DSD is more commonly reported in boars, stallions and dogs (1% – 12%) than in domestic ruminants (< 1%) and in most cases presumed to be inherited (Amann & Veeramachaneni 2007).

Only a few cases of cryptorchidism have been reported in non-domestic ungulates with undefined aetiology, including mule deer (Beauchamp & Jones 1957), Sitka black-tailed deer (Latch et al. 2008), springbok (Skinner 1971) and an Arabian oryx, which also had uterine tissue (Padilla et al. 2005). In goats, cryptorchidism, penis abnormalities and hypospadias are more commonly seen in XY DSD, previously termed male pseudohermaphrodites (King, Young & Fox 2002). Hypospadias is a malformation, in which the urethra opens on the ventral aspect of the penis, in contrast to epispadias, a rare abnormality, where the urethra opens at the dorsal surface of the penis (Lyle 2007; Stephens & Hutson 2005). This article reports a disorder of sexual development in a phenotypic male giraffe (*Giraffa camelopardalis*), with unknown chromosomal type and testicular gonadal sex.

## Ethical considerations

This article followed all ethical standards for research and handling of the animal, demonstrated best practice of veterinary care and occurred in compliance with animal welfare guidelines.

## Case presentation

### History

A 9- to 10-year-old giraffe (*Giraffa camelopardalis giraffa*) in a private game reserve in South Africa was observed to have an abnormal structure ventral to the anus resembling a short penis. Phenotypically, the animal appeared male based on skull formation, horns, body shape and size; however, no external testes were visible.

On 12 March 2018, the animal was darted from a vehicle using 18 mg of thiafentanil (A-3080, Wildlife Pharmaceuticals, Inc., White River, South Africa) administered intramuscularly via a Dan-Inject dart gun. The giraffe was brought into right lateral recumbency by using ropes, blindfolded and its neck placed on a board to maintain the head above the rumen. To decrease the risk of severe hypoxaemia, the giraffe was immediately reversed with 200 mg of naltrexone (Trexonil, Wildlife Pharmaceuticals, Inc., 100 mg intravenously and 100 mg intramuscularly), and manually restrained. Breathing and physiological parameters were monitored until being released 32 min after darting.

### External examination

**FIGURE 1:**
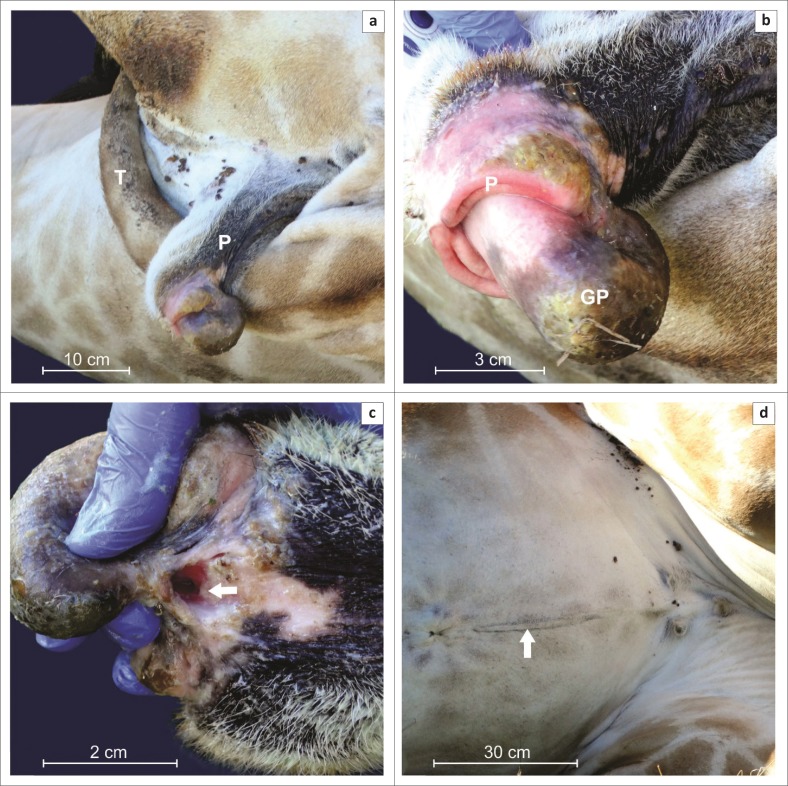
Macroscopic appearance of external genitalia in a giraffe. See tail (T) for orientation of the penis (P) (a). Closer view (b), with structures identified as the prepuce (P) and head of the penis (glans penis, GP). Note the urethral opening (arrow) at the dorsal aspect of the distal penis (c) and the skin depression (arrow) on the ventral midline caudal to the umbilicus (d).

During the external examination, no scrotum was visible or palpable. The structure ventral to the anus was identified as a malformed short penis with a length of 21 cm. The tip was bulbous, pointed caudo-ventrally and had a caudo-dorsal urethral opening, which would have been situated ventrally if the penis would have been situated in its normal position (Figure 1a–c). The mucosa on the distal 5.5 cm of the penis was exposed and showed mucosal ulceration (Figure 1b). No urethral process was present. The surrounding mucosal folds resembled a normal prepuce. Turbid urine dribbled from the urethra and dried urine was present along the caudal aspect of both legs. No spermatozoa were found on microscopic evaluation of the urine. On the ventral midline extending caudal to the umbilicus was a well-circumscribed skin depression (1 cm × 30 cm; Figure 1d).

### Ultrasound

**FIGURE 2:**
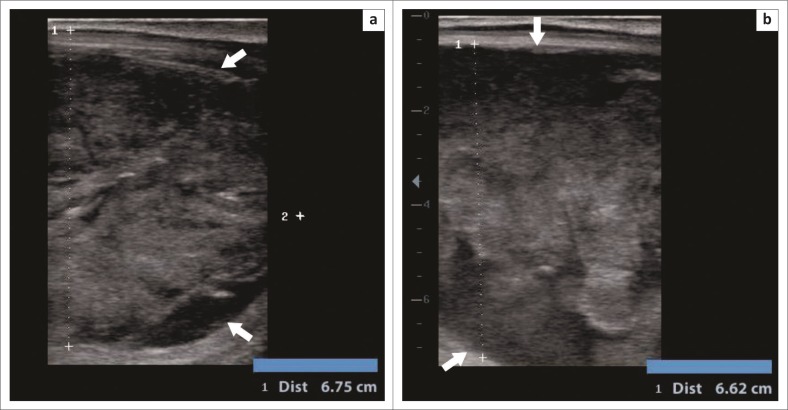
Ultrasound images of a structure ventral to the anus, later identified as a distended vesicular gland (a and b).

A transrectal sonographic examination was conducted by using a portable ultrasound device (mindray DP-50Vet, Shenzhen Mindray Bio-medical electronic Co., LTD., Shenzhen, China) with a linear probe. A circumscribed oval structure approximately 7 cm in height and consisting of irregular tissue was observed ventral to the rectum (Figure 2), but could not be positively identified.

### Blood

A blood sample was collected in serum tubes from the left jugular vein for reproductive hormone measurement. Testosterone and oestrone sulphate concentrations were measured at the Reproduction Laboratory of the Faculty of Veterinary Science, Onderstepoort.

Because of a lack of reference data in giraffes, the concentrations were compared with data collected by the same team from adult wild male giraffes (testosterone: 0.16 nmol/L – 18.94 nmol/L; oestrone sulphate 0.26 n/mL – 0.52 n/mL; unpublished data) and analysed in the same lab. The testosterone concentration was 16.27 nmol/L and the oestrone sulphate concentration was 0.03 ng/mL.

### Post-mortem

The animal was hunter-harvested on 03 August 2018. A post-mortem examination was performed on site and the reproductive organs were transported to the pathology unit of the Department of Paraclinical Sciences, Faculty of Veterinary Science, Onderstepoort, for detailed examination.

#### Pathology

**FIGURE 3:**
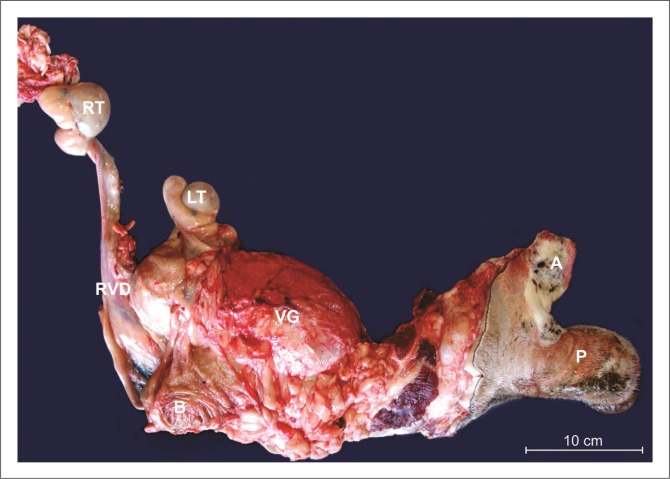
Macroscopic appearance of the abnormal genital tract *ex situ* with the left testis (LT) and right testis (RT), right deferent duct (right vas deferens [RVD]), distended vesicular gland (VG), penis (P), bladder (B) and anus (A).

**FIGURE 4:**
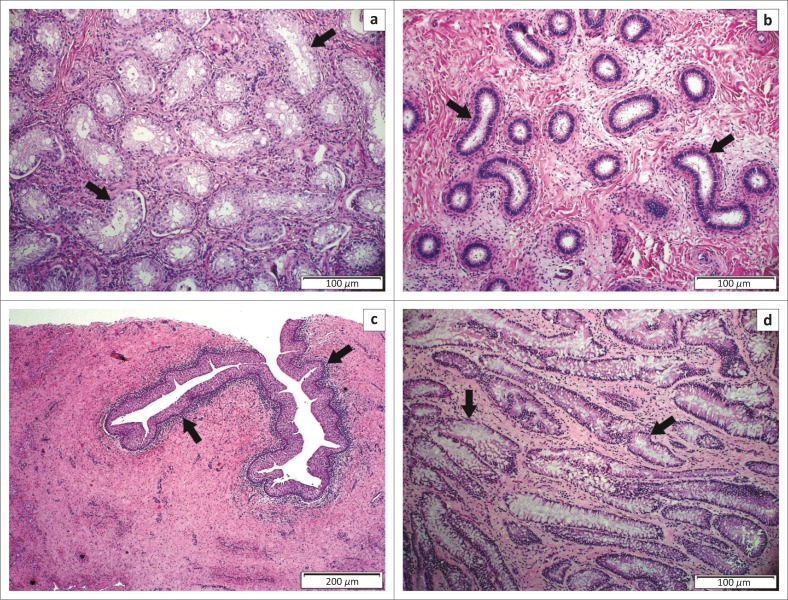
Photomicrographs of (a) the testes, (b) epididymis, (c) deferent duct and (d) vesicular gland. (a) Note small immature seminiferous tubules (arrows) lined by Sertoli cells and the absence of seminiferous epithelium. (b) Epididymis coils (arrows) are small, widely separated by fibrovascular connective tissue and contain no spermatozoa. (c) A layer of neutrophils, lymphocytes and plasma cells (arrows) occurs in the superficial submucosa of the deferent duct. (d) Note the branching tubular glands (arrows) of the deep portion of the vesicular gland.

The testes were situated intra-abdominally and cranio-dorsal to the bladder (Figure 3). The dimensions were 4.3 cm × 3.8 cm × 3 cm (left testicle) and 5.4 cm × 4.0 cm × 3.5 cm (right testicle), excluding the epididymis. The right testis was situated more cranially with a longer deferent duct (approximately 35 cm) than the left (approximately 12 cm). Histologically, both testes consisted of immature tubules lined only by Sertoli cells with small, variably mineralised foci of degenerated and/or necrotic cells in the lumen but no seminiferous epithelium (Figure 4a). The rete testes were normal. Moderate to large numbers of neutrophils diffusely infiltrated the superficial submucosa of the deferent duct (Figure 4c), mixed multifocally with moderate numbers of lymphocytes and plasma cells. Neutrophils also infiltrated the overlying epithelium. The ampullae and spermatic cord vasculature were histologically unremarkable.

The epididymis of both testes was large compared with the testes. Histologically, epididymal tubules were intact with small lumens, widely separated by fibrovascular connective tissue and contained no spermatozoa (Figure 4b).

A large cystic structure dorsal to the rectum measured approximately 12 cm × 12 cm × 25 cm was filled with pale tan mucoid material and identified as a greatly distended vesicular gland (Figure 4d) with mild multifocal acute necrotising neutrophilic inflammation. The other vesicular gland was not identified.

The giraffe was a phenotypic male and presented with a disorder of sexual development with cryptorchidism, hypospadias, penile hypoplasia and abnormal penis positioning.

## Discussion

The external and internal genitalia of this male giraffe showed multiple abnormalities, and the presence of multiple reproductive abnormalities in this giraffe is suggestive of single or multiple genetic defects. The new terminology for the classification of the DSD is followed and the abnormalities are categorised in association with the chromosomal, gonadal and phenotypic sex. This classification was developed in human medicine to replace the term ‘intersex’ (Hughes 2008), and the application, as well as the correlation with the old terminology, has been confusing and inconsistently used in the literature on DSD in animals.

The chromosomal sex of the animal is unknown, because karyotyping was not available. No evidence of a chromosomal DSD was present in this giraffe.

Gonadal sex of the animal was testicular. The testes were situated intra-abdominal, hypoplastic and less ovoid than in normal adult male giraffes (10 cm – 14 cm × 6 cm – 8 cm; Hall-Martin et al. 1978), most likely because of associated hypoplasia and possible secondary degeneration of the seminiferous epithelium as a result of the effect of body temperature on this epithelium (Kim, Park & Rhee 2012). Although testicular neoplasia is common in retained testes (Amann & Veeramachaneni 2007), no evidence of neoplasia was found in this giraffe.

The deferent duct, particularly the left one, was shorter than that described in normal adult male giraffes (approximately 60 cm; Hall-Martin et al. 1978). The inflammation of the deferent duct and vesicular gland was likely an ascending infection because of lack of normal anatomical protection (Cavalieri & Van Camp 1997). The distended vesicular gland may have occurred because of blockage by physical factors or inflammation or as a result of a mesonephric duct anomaly possibly resulting in fusion of the two vesicular glands as has been described for bulls (Bagshaw & Ladds 1974; Cavalieri & Van Camp 1997).

Phenotypically, the external genitalia more closely resembled male than female organs. The penis was shorter than in normal male giraffes (up to 77 cm when flaccid; Hall-Martin et al. 1978), situated in the perineal area and directed caudally. If the penis was in its normal position in the ventral midline, the urethra would have opened on the ventral surface (hypospadias). Malformations of the penis are rare in all species and include a short penis, lack of sigmoid flexure, hypospadias and epispadias in domestic ruminants (Saunders & Ladds 1978; Stephens & Hutson 2005), but have not been reported in wild ungulates.

The cause and identity of the DSD in this giraffe are undetermined. Cryptorchidism, penile hypoplasia and hypospadias without the presence of female genitalia in dogs may result from incomplete masculinisation of the internal and external genitalia (Lyle 2007). However, similar abnormalities have also been reported in XX dogs with gonadal DSD (XX sex reversal syndrome; Switonski et al. 2011) and in XY goats identified with phenotypic DSD (male pseudohermaphrodites; Eaton 1943). No uterine or ovarian tissue was present in this giraffe, and testosterone blood concentrations were within the range measured in sexually active males (0.5 nmol/L – 64.01 nmol/L, unpublished data). The normal testosterone concentrations but partial failure of masculinisation of the genitalia might indicate androgen receptor defects in this giraffe, as described for dogs and cats (Lyle 2007). Oestrone sulphate was considerably lower than detected in wild male giraffes (range 0.26 ng/mL – 0.52 ng/mL, unpublished data). Lower oestrone sulphate concentrations were also reported in cryptorchid horses compared with intact stallions (Carneiro et al. 1998).

In wildlife, DSD are likely to be underestimated because these species are not as closely monitored as domestic ones (Mastromonaco, Houck & Bergfelt 2012). Additionally, existing cases might not be examined because of cost involved and lack of necessity. Although DSD are likely to occur rarely in free-ranging animals because of natural selection, in-breeding is known to increase the risk for congenital defects (Brown et al. 2009). This giraffe was part of a small, isolated and possibly in-bred population, but random chromosomal mutations, environmental factors or exposure to endocrine disrupters cannot be excluded. The latter have been linked to outbreaks of cryptorchidism (Amann & Veeramachaneni 2007). The separation of the penis-like structure from the ventral abdominal wall and the bulbous tip to the penis with missing urethral process could indicate a traumatic origin or contribution to the penile lesion but does not account for the changes in the rest of the reproductive tract.

Disorders of sexual development have not been previously described in giraffes. Discovery and reporting of other cases in giraffe and other wild ruminants might help to identify the underlying causes and importance of these abnormalities for the reproductive potential of wildlife populations.
